# Next steps in eliminating tuberculosis in the Americas

**DOI:** 10.1016/j.lana.2023.100512

**Published:** 2023-05-15

**Authors:** The Lancet Regional Health – Americas

Globally, tuberculosis is the leading cause of death by infectious disease, topped only by COVID-19. Elimination of tuberculosis by 2030 is one of the Sustainable Development Goals. As an airborne disease that has infected about a quarter of the global population, tuberculosis control as a specific goal related to good health and wellbeing recognises that this is an international humanitarian crisis. Groups at high risk include homeless people, healthcare professionals, immigrants, Indigenous people, and incarcerated people. However, from the beginning of the COVID-19 pandemic, the challenge to eliminate tuberculosis became even more prominent. The upsurge of cases and deaths affected many locations, including the Americas and the Caribbean. According to Pan American Health Organisation (PAHO), this region represented 3% of the global burden in 2020.

Although the Americas is not the region with the highest burden, tuberculosis still has a major impact on the health and quality of life of 291 thousand people. Brazil accounts for 33% of cases in the region, followed by Peru with 13.1%, and Mexico with 10.7%. To eliminate tuberculosis by 2035, Brazil, one of the 30 countries with the highest burden of tuberculosis in the world, established three main goals: to reduce tuberculosis incidence by 90%, to reduce mortality by 95% in relation to 2015, and to reduce the number of people affected by catastrophic costs due to tuberculosis by 2035. Before the COVID-19 pandemic, Brazil failed to reach the WHO's targets for tuberculosis elimination, and during the first 2 years of COVID-19 pandemic, the targets became more challenging. Tuberculosis is a compulsory notifiable disease in Brazil, but the number of undiagnosed and untreated patients increased during the COVID-19 pandemic, and it is now one of the 16 countries with tuberculosis undernotification. In the past 20 years, a decrease in mortality was observed in Brazil, but the pandemic reversed this trend, with 5072 notified deaths in 2021 compared with 4569 in 2020. One of the major epidemiological changes was the 10.9% increase in cases in children aged 0–14 years in 2022. This could be related to the low uptake of the BCG vaccine in the population and the exposure to domiciliary tuberculosis infections during the lockdown period. A 93% notification reduction was observed in 2021, and according to Coutinho and colleagues, the second wave of the COVID-19 pandemic was characterised by a sharp drop in tuberculosis preventive treatment (TPT) prescriptions, higher than the decrease in tuberculosis disease notifications. Another country of concern is Peru, which has a high burden of multidrug-resistant tuberculosis. In Peru during the COVID-19 pandemic, tuberculosis-related deaths increased 3.3 times, and detected tuberculosis cases decreased by 8000 compared with 2019 because of undernotification and reduced screening.

In 2019, a *Lancet* Series on Tuberculosis elimination was launched. One key aspect of tuberculosis elimination is an efficient diagnosis and treatment retention for transmission to be effectively stopped. By accentuating inequalities, the COVID-19 pandemic set us back years with the disruption of tuberculosis services and notifications and direct consequences in the cascade of care. However, one must point out the progress related to tuberculosis diagnosis and treatment in the years preceeding the COVID-19 pandemic. In Peru, a programme focussing on the tuberculosis cascade-of-care, combining molecular diagnosis, screening comorbidities, and monitoring systems (Sistema de Información Gerencial de Tuberculosis) had some success before COVID-19. As two infectious diseases that are a threat to populations worldwide, strategies to screen for both COVID-19 and tuberculosis are welcome. This strategy was successfully implemented in a study by MacLean and colleagues that integrated COVID-19 and tuberculosis testing in a single sputum sample in Peruvian patients, identifying 96% of tuberculosis and 67% of COVID-19 cases, a promising method to diagnose both diseases at the same time. An update in approaching treatment dropouts and loss to follow-up are also urgently needed, such as telemedicine, home visits, and SMS monitoring. Other effective strategies to eliminate the disease include TPT and adherence to tuberculosis infection treatment. One example is the recommendation by the Brazilian Ministry of Health to provide TPT to those in high-risk groups. On April 17, 2023, a decree to eliminate tuberculosis in Brazil was established, a welcome change in the progress of tuberculosis elimination in the country. For these actions to be properly implemented, investments are necessary, as in past years allocated resources have decreased, with a direct impact on tuberculosis control.

People at high risk of tuberculosis need to be targeted and interventions are necessary to eliminate disparities in a cost-effective way. For example, tuberculosis incidence is ten times higher in prisons than in the general population, explained by the paucity of medical access, overcrowding and smoking, and the presence of comorbidities. Additionally, the use of the sputum smear technique, which has a 10% sensitivity in children and 30% in people living with HIV, as main diagnostics in many middle-income and low-income countries fails to detect many infections. Ramon Soares and colleagues proposed a chest-ray computer-aided detection interpretation algorithm as an initial screening tool for tuberculosis in prisons in Brazil that met the WHO Target Product Profile requirements for a triage test in high-burden locations. Comorbidities, such as diabetes, are also a risk factor for tuberculosis, and the combination of reporting and screening for both diabetes and tuberculosis could be a useful strategy for countries with highest incidence of diabetes, such as Mexico and the USA.

Even though it is a preventable, treatable, and curable disease, the key drivers of the tuberculosis pandemic, such as poverty, diabetes, undernourishment, HIV status, and tobacco and alcohol use, need to be addressed and accounted for. Despite the advances made in the Americas, the COVID-19 pandemic interrupted and disrupted most of the progress, and inequalities are even more prominent. The paucity of funding and disruption in tuberculosis notification and treatment puts tuberculosis back on the radar in the Americas, and a stronger commitment is necessary. There is an urgent need to address inequities when dealing with tuberculosis elimination, as those who are in vulnerability are at higher risk. As Dr Tedros Adhanom (WHO) pointed: “(…) with solidarity, determination, innovation and equitable use of tools, we can overcome severe health threats. Let's apply those lessons to tuberculosis. It is time to put a stop to this long-time killer. Working together, we can end tuberculosis.”

